# Testing the Factor Structure and Construct Validity of the German Version of Gray et al.’s (2003) Sexual Fantasy Questionnaire

**DOI:** 10.1007/s10508-024-02831-7

**Published:** 2024-03-21

**Authors:** Robert J. B. Lehmann, Thomas Schäfer, Ross Bartels, Sabina Sabic, Catrin Schache

**Affiliations:** 1 Department of Psychology, MSB Medical School Berlin, Rüdesheimer Straße 50, 14197 Berlin, Germany; 2 School of Psychology, University of Lincoln, Lincoln, UK

**Keywords:** Sexual fantasy, Sex drive, Sexual sensation-seeking, Sexual compulsivity, Sexual excitation, Sexual embarrassment

## Abstract

Gray et al.’s (2003) Sexual Fantasy Questionnaire (SFQ) is becoming an increasingly used self-report measure of sexual fantasy use. The current study analyzed the factorial structure and construct validity of the behavioral items of the SFQ using a nomological network of other sexuality-related measures in a large German-speaking sample (*N* = 846). Participants’ (27.7% females) mean age was 30.8 years (*SD* = 11.0). Exploratory factor analysis revealed a 65-item scale comprising five-factors, which were termed: normophilic sexual fantasies, sexualized aggression, sexualized submission, submissive courtship, and bodily functions. This German version of the SFQ was found to have high construct validity indicated by its association with other related constructs. Based on these results, we argue that the SFQ is a valid self-report measure that can be used in both research and clinical practice (foremost the factors sexualized aggression and sexualized submission). Suggestions for future research are discussed in light of the results and the study’s limitations.

## Introduction

Sexual fantasizing refers to the (often deliberate) cognitive act of mentally envisioning a sexual scenario (Bartels et al., 2021). It is regarded as a key aspect of human sexual functioning as it can help to induce and/or increase sexual arousal (Davidson, 1985). This is because people tend to sexually fantasize about content they find sexually arousing (Leitenberg & Henning, 1995), whether it be a behavioral category (e.g., intimate, impersonal), a specific behavior (e.g., oral sex), a target category (e.g., men, women), and/or a specific target (e.g., partner, celebrity) (Joyal et al., 2015; Wilson, 1978). Sexual fantasizing can also serve other functions, such as regulating emotional states (Gee et al., 2003), increasing one’s engagement in relationship-promoting behaviors (Birnbaum et al., 2019), and indulging (mentally) in sexual scenarios that one does not wish to (or cannot) experience in real-life (Joyal et al., 2015; Willis & Bartels, 2024; Wilson, 1978). It can also motivate sexual behavior, supported by research showing that sexual fantasizing is associated with the corresponding behavior (Noorishad et al., 2019). This is not an issue for people who wish, for example, to engage in more varied sexual behaviors with a consenting partner. However, it can be an issue if the fantasy content involves, for example, sexual activity with children (Dombert et al., 2016) or sexual coercion (Bondü & Birke, 2020) as these acts would constitute a sexual offense if enacted. Indeed, research shows that the use of such fantasies not only correlates with sexual arousal towards the same behavior (Bártová et al., 2021) but also with actual engagement in the corresponding behavior (Klein et al., 2015; Williams et al., 2009). This is because sexual fantasizing can increase sexual motivation (Smid & Wever, 2019) and can help model future offending behavior (Gee et al., 2003). It should be noted, however, that the link between sexual fantasizing and offending behavior is affected by other factors (Klein et al., 2015; Williams et al., 2009; Willis & Bartels, 2024). Thus, not everyone will enact a fantasy involving offending behavior. Nevertheless, sexual fantasizing is regarded as both an important factor to consider in sex offending theories (e.g., Ward & Beech, 2006) and as a target for treatment in forensic settings (Allen et al., 2020). Given the various effects and functions that sexual fantasizing can have, it is important that researchers and clinicians have a reliable and valid measure for assessing how often people use sexual fantasies.

Sexual fantasy use is primarily measured using questionnaires. They involve presenting a list of different sexual acts and targets that are rated in terms of how frequently they are fantasized about. These ratings tend to be summed and converted into sexual fantasy themes (e.g., intimate, sadomasochistic, impersonal), typically determined via factor analytic methods. These themes are helpful as they enable assessors to reduce the vast number of fantasy items down to a more manageable number of composite variables. They are also meaningful variables. That is, the fact that a disparate set of sexual fantasy items comprise a single category may suggest that the fantasy items are underpinned by a common feature, mechanism, or function. Thus, the use of fantasy themes can help refine our theoretical understanding of sexual fantasy use and help establish where similarities and differences exist between subgroups.

Internationally, different sexual fantasy questionnaires have been introduced into the literature, such as the Wilson Sex Fantasy Questionnaire (WSFQ; Wilson 1978), Male Sexual Fantasy Questionnaire (MSFQ; Smith & Over, 1991), Female Sexual Fantasy Questionnaire (FSFQ; Meuwissen & Over, 1991), O’Donohue et al.’s (1997) Paraphilic Sexual Fantasy Questionnaire, and the Sexual Fantasies and Behaviors Inventory (Brown et al., 2022). Of these, the most long-standing, commonly used, and best-validated measure is the 40-item WSFQ (Bartels et al., 2019). However, this scale is said to contain some outdated, vague, and non-sexual items (Brown et al., 2022; Joyal et al., 2015), and also combines sadistic and masochistic items under one factor. To address this, the scale was modified and expanded (55-items) by Joyal et al. (2015), which Dyer and Olver (2016) referred to as the Joyal Sexual Fantasy Questionnaire (JSFQ). This version made reference to the sex of the target in some of the items. As a result, the JSFQ was found, by Dyer and Olver (2016), to have three target-specific factors (i.e., female-focused, male-focused, and anonymous) across various behaviors. The other items loaded onto three factors reflecting broad behavioral categories (i.e., sexualized dominance, paraphilias, and non-coital sexual activities). Like the sadomasochistic factor of the WSFQ, the eroticized dominance factor (primarily) contained both dominant and submissive items. Thus, differences between these two fantasy themes would be obscured when comparing subgroups of people (e.g., men vs. women, people who have offended vs. nonoffenders) on this combined factor. In contrast, Brown et al. (2022) found distinct factors for these two fantasy themes when examining the structure of their Sexual Fantasies and Behavior Inventory (SFBI). However, their measure was “limited in its range of sexual fantasies” (Brown et al., 2022, p. 228). It also assessed respondents’ subjective arousal towards each fantasy item, as opposed to its frequency of use. Similarly, the JSFQ assesses the “intensity” of each fantasy item. As a result, it may be difficult to directly compare the JSFQ and SFBI to other measures that assess frequency of fantasy use.

One measure that assesses frequency of sexual fantasy use is the Sexual Fantasy Questionnaire (SFQ; Gray et al., 2003). The SFQ consists of 93 items, six of which refer to binary sexual orientation targets (i.e., adult males/females, adolescent males/females, pre-pubescent males/females). The other 87 items refer to specific sexual behaviors. As noted by Gray et al. (2003), “15 sadistic fantasy items were included in the questionnaire along with 72 filler items relating to other types of sexual fantasy (e.g., intimate, exhibitionistic, fetishistic, masochistic)” (p. 1023). Thus, although designed to assess the use of sexually sadistic fantasies, the SFQ contains many other items across a diverse range of themes. As such, it possesses sufficient variation to be used as a general measure of sexual fantasy use and has been gaining increased interest from researchers (Deehan & Bartels, 2021; Harvey & Jeglic, 2020; Spada & Jeglic, 2016). However, the original authors did not examine or provide information regarding the factor structure and psychometric properties of the SFQ.

To address this, Bartels and Harper (2018) explored the factor structure of SFQ using the amended 5-point Likert scale proposed by Maile (2015) and the 87 behavioral items (not the six target-specific items). Only items with a factor loading above 0.40 were retained as only these are suitable to represent a specific latent variable (Field, 2005; Stevens, 2012). They initially found a 6-factor solution using principal components analysis. However, they later reanalyzed the data in 2021 using the Maximum Likelihood extraction method (see https://osf.io/bt5dm). This ultimately resulted in a five-factor solution, comprising 59 of the 87 items. The five factors were labeled Romantic, Impersonal, Sexualized Aggression, Sexualized Submission, and Bodily Functions. In contrast to the WSFQ and JSFQ, aggressive/dominance fantasies and submissive fantasies loaded on distinct factors, similar to Brown et al. (2022). The factors showed good to excellent internal consistencies (alphas ranging from 0.73 to 0.97). Given its factorial validity and shorter length, Bartels and Harper proposed that this revised 59-item version of the SFQ can be used as an alternative to the original.

### Current Study

The factorial structure of the SFQ has not been examined in other countries, nor has its construct validity been tested. Establishing such validity will help support the revised SFQ as a measure of sexual fantasy use. Therefore, the aim of the present study was to analyze the factorial structure of the SFQ in a sample of German participants, and to provide a test of its construct validity (i.e., convergent and discriminant validity). Convergent validity refers to whether a measure of one construct is associated with other measures of the same or related constructs. With regards to sexual fantasizing, similar and related constructs would include sex drive, sexual sensation-seeking, sexual compulsivity, and sexual excitation, as they involve experiencing frequent sexual thoughts and/or a preoccupation with sexual behavior. Conversely, sexual fantasizing should be different from constructs such as sexual inhibition, sexual embarrassment, sexual self-focus, and dysfunctional sexual beliefs, so that we expected small or zero correlations with these variables to ascertain discriminant validity.

According to Baumeister et al. (2001) “sexual fantasies are probably one of the best indexes of strength of sex drive because they are explicitly sexual and require conscious attention but are not constrained by opportunities, social pressures, or other external factors” (p. 246). As a person with a strong sex drive would likely also often think about sex, we would expect a positive relationship between sexual fantasy use and sex drive levels (Wilson, 1978).

Sexual sensation-seeking constitutes the dispositional need for varied, novel, and complex sexual experiences and can be assessed by the Sexual Sensation Seeking Scale (Kalichman & Rompa, 1995). The scale has been found to correlate with sexual fantasy use (Marker & Schneider, 2015; Seifert et al., 2017), as well as the number of one-night-stand sexual encounters (Gaither & Sellbom, 2003; Hendershot et al., 2007) and varied sexual practices (Gutiérrez-Martínez et al., 2007). Thus, we would expect people scoring high on sexual sensation-seeking to fantasize more frequently and about more varied sexual experiences (e.g., impersonal sexual fantasies).

Sexual compulsivity is characterized by insistent, intrusive, and uncontrolled sexual thoughts and behaviors and can be assessed with the Sexual Compulsivity Scale (Kalichman et al., 1994). Research shows that sexual compulsivity is correlated with sexual fantasy use (Dyer & Olver, 2016), a greater number of sexual partners, and lower sexual control (Kalichman & Rompa, 1995; Kalichman et al., 1994), as well as internet use for sexual purposes and the likelihood to seek sex partners in anonymous sexual exchange venues and clubs (Cooper et al., 1999; Dodge et al., 2008). Given the insistent sexual thoughts and behavioral correlates of sexual compulsivity, we expected to find a positive relationship with sexual fantasy use (e.g., impersonal sexual fantasies).

Furthermore, we were interested in the relationship between sexual fantasy use and sexual excitation. According to Bancroft and Janssen (2000), sexual arousal and related processes result from a balance between inhibitory and excitatory mechanisms. Individuals with an unusually high propensity for excitation or a low propensity for inhibition are more likely to engage in high-risk or otherwise problematic sexual behavior, whereas individuals with a low propensity for sexual excitation or a high propensity for sexual inhibition are more likely to experience problems with impairment of sexual response (Bancroft et al., 2009). The Sexual Inhibition/Sexual Excitation Scales (SIS/SES) assess the propensity for sexual inhibition and excitation (Janssen et al., 2002a, 2002b) and have been found to be related to sexual desire, sexual arousal, sexual risk-taking, sexual compulsivity, and hypersexuality (Bancroft et al., 2009). Walton and Bhullar (2018) found the SES to be moderately correlated with sexual fantasy use. Therefore, we would expect a positive relationship between SFQ scores and sexual excitation, but a negative relationship between sexual fantasy use and sexual inhibition.

In relation to sex-related affect, we included sexual self-consciousness (i.e., the propensity to become self-conscious in sexual situations) in our nomological network. This can be assessed using the Sexual Self-Consciousness Scale (van Lankveld et al., 2008), which consists of two subscales termed “Sexual Embarrassment” and “Sexual Self-Focus.” We would expect people with increased feelings of embarrassment, uncertainty, and discomfort in sexual situations to engage in less sexual fantasizing indicated by a negative effect. In addition, we hypothesized that dysfunctional sexual beliefs would also be negatively associated with sexual fantasizing. Pascoal et al. (2017) developed the Beliefs about Sexual Function Scale to enable researchers to determine the specific role of beliefs about sexual functioning on sexual outcomes. This included five sets of beliefs pertaining to anal sex, male performance, aging, sexual pain, and primacy of the relationship. Validation research on this measure is limited. However, Pascoal et al. (2018) found a small negative relationship with sexual functioning. Thus, we would also expect a negative association.

To test the construct validity of the SFQ, we used a nomological network (Cronbach & Meehl, 1955). We sought to elaborate the nomological network of the SFQ with an explicit focus on the sexuality-related constructs described above. Defining expected positive and negative relationships with other sexuality-related measures based on previous empirical findings will aid in testing the construct validity of the SFQ (Campbell & Fiske, 1959).

## Method

### Participants

Our initial sample comprised 891 adult community members, recruited online via social media platforms (e.g., Reddit, Facebook, Instagram) between 24 June 2021 and 15 August 2021. Of those participants, we excluded three participants younger than 18 years old, nine participants who failed the seriousness check by answering that they had not taken part seriously (Aust et al., 2013), and 33 participants who failed the honesty check by indicating they had answered dishonestly more than five times in the questionnaire (Sischka et al., 2022). This resulted in a final sample of 846 participants. The mean age of the sample was 30.8 years (*SD* = 11.0, range 18 to 80). In 2022, 24.5% of the German population were 20 to 40 years old (Statistisches Bundesamt, 2023; with 74.0% being between 20 and 80 years old). The majority (69.6%) identified as a man, with 27.7% identifying as a woman, and 2.7% as minority otherwise identities (predominantly as non-binary or trans). Most (43%) were university students or had a university degree, 37.8% completed the German Abitur, 17% held a degree lower than abitur, and 2.1% provided no answer. In 2018, more than half (56%) of the population of Germany aged 25 and over had a higher-level school-leaving qualification (Statistisches Bundesamt, 2021). Among the 25 to 29-year-olds, 80% had such a qualification (27% with an intermediate qualification, 53% with an Abitur).

### Measures

#### Sexual Fantasy Questionnaire

The Sexual Fantasy Questionnaire (SFQ) by Gray et al. (2003) consists of 93 items. Six items refer to sexual orientation targets (i.e., adult males/females, adolescent males/females, pre-pubescent males/females), while the other 87 refer to specific sexual behaviors. Items were rated using Maile’s (2015) 5-point response format (1 = “Have never fantasized about” to 5 = “Have fantasized about very frequently”). For translation of the questionnaire, we followed a committee approach (European Social Survey, 2014).

#### Sex Drive Scale

The Sex Drive Scale by Lippa (2006) includes the following five items: (1) I have a strong sex drive, (2) I frequently think about sex, (3) It doesn’t take much to get me sexually excited, (4) I think about sex almost every day, and (5) Sexual pleasure is the most intense pleasure a person can have. Items were rated using a 7-point Likert response format ranging from 1 (do not agree at all) to 7 (completely agree). Reliability analysis showed a good consistency index (α = 0.87). Scores were averaged for analysis.

#### Sexual Sensation Seeking Scale

The Sexual Sensation Seeking Scale (SSSS) was developed to measure the propensity to seek exciting, risky, or novel levels of sexual stimulation or arousal (Hammelstein, 2005; Kalichman et al., 1994). The 11 items were rated using a 4-point response format ranging from 1 (not at all like me) to 4 (very much like me). Scores were averaged for analysis. Reliability analysis showed a good consistency index (α = 0.81).

#### Sexual Compulsivity Scale

The Sexual Compulsivity Scale (SCS) was developed to measure sexually compulsive behaviors, sexual preoccupations, and sexually intrusive thoughts (Hammelstein, 2005; Kalichman & Rompa, 1995). The 10-items were rated using a 4-point response format ranging from 1 (not at all like me) to 4 (very much like me). In the present study, items were averaged for analysis, and showed a good Cronbach’s alpha (α = 0.87).

#### Sexual Inhibition and Sexual Excitation Scales

The Sexual Inhibition and Sexual Excitation Scale (SIS/SES) (Janssen et al., 2002a, 2002b; Rettenberger & Briken, 2013) is based on the dual-control model of sexual arousal (Bancroft & Janssen, 2000) and comprises three subscales: (1) sexual excitation (SES, 6 items), (2) sexual inhibition due to threat of performance failure (SIS1, 4 items), and (3) sexual inhibition due to threat of performance consequences (SIS2, 4 items). The 14-items were rated using a 4-point Likert response format from 1 (“strongly agree”) to 4 (“strongly disagree”). Scores were averaged for analysis. The internal consistency in the present study was α = 0.78 for SES, α = 0.58 for SIS1, and α = 0.59 for SIS2.

#### Sexual Self-Consciousness Scale

The Sexual Self-Consciousness Scale (SSCS) was designed by van Lankveld et al. (2008) to assess individual variability with regard to the propensity to become self-conscious in sexual situations. The SSCS consists of 12 items forming the two subscales of sexual embarrassment (items 1–6) and sexual self-focus (items 7–12). Items were rated on a 5-point Likert response format (1 = Strongly disagree to 5 = Strongly agree). Scores were averaged for analysis. For translation of the questionnaire, we followed a committee approach (European Social Survey, 2014). The internal consistency in the present study was α = 0.84 for sexual embarrassment and α = 0.70 for sexual self-focus.

#### Beliefs About Sexual Functioning Scale

The Beliefs About Sexual Functioning Scale (BASEF) was designed by Pascoal et al. (2017) to assess dysfunctional beliefs about sexual functioning. The BASEF consists of 15 items forming five factors (anal sex beliefs, sexual pain beliefs, male performance beliefs, aging beliefs, and primacy of the relationship beliefs), which can be aggregated into a single latent factor. Items were rated using a 5-point Likert response format (1 = totally disagree to 5 = totally agree). Scores were averaged for analysis. For translation of the questionnaire, we followed a committee approach (European Social Survey, 2014). Reliability analysis showed an acceptable consistency index (α = 0.78).

### Procedure

Participants were recruited online using different German-language platforms (e.g., Reddit, Facebook, Instagram). At the beginning of the study, all participants were informed about the content of the study (e.g., sexual fantasies), as well as the expected duration (20 to 30 min). Subsequently, participants were informed about inclusion and exclusion criteria. Also, the anonymity of the participants was assured, and it was pointed out that the survey could be terminated at any time. Any person was allowed to participate, who could confirm that he or she was at least 18 years old. Participants had to provide informed consent before starting the study. At the end of the study, the aims were explained to the participants in a debriefing.

## Results

### Data Preparation

Across all items, a maximum of 1.9% of missing values were observed. The missing values did not reveal any recognizable pattern, as indicated by visual inspection and Little’s MCAR test (*p* > 0.999). Therefore, the missing values were replaced using the Expectation Maximization algorithm, using all available metrically scaled data from the questionnaires, so that a complete data set with *N* = 846 was available for analysis.

### Confirmatory Factor Analysis

We first ran a confirmatory factor analysis (CFA) to see if the data from our German sample fit the five-factor model obtained by Bartels and Harper (2018, 2021). However, the fit turned out to be insufficient: χ^2^ = 11,745, *p* < 0.001, χ^2^/*df* = 7.2, *GFI* = 0.63, *CFI* = 0.66, *TLI* = 0.65. Thirteen items had factor loadings below 0.5. Thus, with the data of our German sample, the five-factor model could not be satisfactorily supported, so a new exploratory search for a latent factor structure was indicated. Thus, in a next step, we used an exploratory factor analysis (EFA) to investigate which factor structure can be derived from our data and to what extent this is comparable to the factor structure found by Bartels and Harper (2018, 2021).

### Exploratory Factor Analysis

An EFA using principal axis factoring was run with all 87 behavioral items. Parallel analysis and the screeplot were used to identify a reasonable number of factors. The data showed high suitability for factor analysis: *MSA* = 0.90. Parallel analysis yielded 15 factors with variance explained above the level of simulated data. However, the 15-factor solution was not useful because many factors consisted of only two or three items, some items had very low factor loadings and communalities, and the solution lacked interpretability. The screeplot, on the other hand, suggested a five-factor solution with much better interpretability with 36% of the overall variance explained. After oblique rotation (direct oblimin), factors showed very low correlations with each other (< 0.30). Because of these low correlations and the better interpretability and greater statistical parsimony of an orthogonal solution (Kieffer, 1998), we used the orthogonal factors. Twenty-two of the 87 items had factor loadings below 0.4 and were removed to arrive at a usable structure. This increased the variance explained to 46%. Table 1 shows the factors and factor loadings of the solution with 65 items (87 minus 22) and five factors.

**Table 1:** Descriptive statistics of the Sexual Fantasy Questionnaire items and the five factors and factor loadings as a result of the exploratory factor analysis with 65 items

	*M*	*SD*	Normophilic fantasies	Sexualized aggression	Sexualized submission	Submissive courtship	Bodily functions
17. Receiving oral sex	3.84	1.21	.627				
19. Touching intimate places	4.10	1.01	.619				
79. Viewing pornographic pictures	2.95	1.41	.613				
76. Sex whilst watching soft pornography	2.42	1.23	.613				
12. Masturbating in front of another person	2.69	1.29	.612				
16. Giving oral sex	3.73	1.27	.611				
80. Being watched whilst having sex	2.28	1.30	.606				
13. Being masturbated by another person	4.06	1.03	.605				
77. Sex whilst watching hard pornography	2.40	1.32	.602				
75. Watching others having sex	2.39	1.24	.593				
8. Sex in public places	3.12	1.24	.559				
91. Using sex toys, (e.g., dildos, inflatable dolls)	3.22	1.28	.559				
56. Sex with multiple partners	3.26	1.23	.547				
89. Exposing your genitals to somebody	1.84	1.16	.537				
74. Being secretly observed by somebody	1.75	1.08	.535				
90. Somebody exposing themselves to you	2.31	1.36	.527				
73. Secretly observing or peeping at somebody	1.97	1.14	.518				
78. Reading pornographic stories	2.66	1.37	.516				
60. Sex in toilets with strangers	2.17	1.24	.515				
15. Anonymous sexual contact with a stranger	3.03	1.34	.501				
7. Sex in beautiful places	3.49	1.16	.485				
58. Anal intercourse	3.21	1.45	.466				
14. Romantic sexual contact with somebody you know	3.75	1.23	.466				
18. Passionate kissing	3.64	1.23	.465				
54. Sex with a drunk person	2.11	1.20	.462				
53. Sex whilst drunk	2.26	1.19	.442				
93. Wearing sexy underwear	2.95	1.39	.425				
24. Physically hurting the person you are having sex with	1.19	1.08		.778			
45. Humiliating somebody	1.65	1.08		.737			
68. Forcing somebody to have sex against their will	1.47	.93		.734			
43. Raping somebody	1.46	.87		.730			
32. Torturing others	1.41	.87		.710			
25. Physically injuring the person you are having sex with	1.28	.74		.707			
28. Strangling or asphyxiating others (without killing)	1.78	1.13		.682			
47. Scaring somebody	1.22	.72		.674			
63. Controlling others	2.07	1.20		.663			
66. Forcing somebody to do something non-sexual against their will	1.26	.67		.648			
36. Whipping somebody else	1.61	1.01		.644			
61. Physically attacking someone	1.17	.57		.643			
51. Drugging or sedating another person for sexual reasons	1.30	.76		.602			
70. Sex while threatening someone with a weapon	1.09	.45		.555			
31. Spanking others	2.20	1.29		.546			
50. Tying up somebody	2.54	1.24		.529			
71. Stalking or secretly following somebody	1.21	.57		.459			
26. Being physically hurt	1.79	1.10			.799		
35. Being whipped on non-sexual parts of the body	1.45	.87			.748		
30. Being spanked	1.88	1.22			.707		
33. Being tortured	1.37	.82			.693		
64. Being controlled	2.05	1.23			.690		
34. Being whipped on sexual parts of body (breasts or genitals)	1.30	.78			.676		
49. Being bound or tied up	2.52	1.27			.672		
46. Being humiliated	1.61	1.07			.654		
27. Being strangled or asphyxiated (without dying)	1.55	1.01			.633		
48. Being scared by somebody	1.21	.67			.631		
65. Being forced to do something non-sexual against your will	1.27	.71			.598		
62. Being physically attacked	1.18	.56			.586		
67. Being forced to have sex against your will	1.55	.98			.582		
44. Being raped	1.54	.90			.508		
69. Sex while being threatened with a weapon	1.13	.51				.520	
72. Being stalked or secretly followed by somebody	1.17	.56				.509	
86. Receiving threatening phone calls	1.04	.31				.442	
39. Having a partner urinate (piss) on you	1.67	1.14					.687
40. Urinating (pissing) on others	1.58	1.04					.651
41. Having a partner defecate (shit) on you	1.07	.36					.530
42. Defecating (shitting) on a partner	1.06	.34					.518

The item numbers are the original item numbers of Gray et al.’s (2003) Sexual Fantasy Questionnaire

Interestingly, the first factor contains items that were part of the romantic factor and the impersonal factor identified by Bartels and Harper (2018, 2021). Since all these items reflected non-paraphilic content, we termed this factor normophilic sexual fantasies. This aligns with the DSM-5’s normophilic versus paraphilic classification system, which, according to Brown et al. (2022, 2023), avoids labeling sexual fantasy content in frequency terms (typical vs. atypical), offending terms (deviant vs. non-deviant), or culturally-bound terms (normative vs. non-normative). The second factor is a perfect replication of the 17-item sexualized aggression factor by Bartels and Harper. The third factor includes 14 items all from the sexualized submission factor by Bartels and Harper. The fourth factor comprises three items reflecting a sexualized submission variant of courtship disorder (Freund & Blanchard, 1986) and so was named submissive courtship. The fifth factor is a perfect replication of the bodily functions factor found by Bartels and Harper (2018, 2021) and was named accordingly. Thus, the results showed that, in an online sample of German-speaking participants, we were able to replicate three out of five factors previously identified by Bartels and Harper (Factors 2, 3, and 5). Factor 1 comprised items from two separate factors found by Bartels and Harper, while Factor 4 included two items of the sexualized submission factor identified by Bartels and Harper (2018, 2021).

### Nomological Network

Table 2 shows the descriptive statistics and reliabilities for all measures used in the present study, and Table 3 shows the Pearson’s correlations between the five SFQ factors and the scales used to analyze its construct validity. As outlined above, we expected positive correlations between sexual fantasy factors and other sexuality-related measures indicative of approach motives of sexual behavior. As hypothesized, for sexual sensation seeking and sexual excitation, results indicated small to large positive relationships with the five sexual fantasy factors, whereas the relationships with sexual compulsivity were small to moderate. Also, we expected a positive relationship between sexual fantasies and sex drive, which was supported for four of five factors (except for sexualized submission), with effect sizes ranging from small to large.

**Table 2:** Descriptives (*M* and *SD*) and reliability (Cronbach’s alpha) of all measures

	No. of Items	*M*	*SD*	α
Sex Drive Scale	5	5.26	1.31	.88
Sexual sensation seeking scale	11	2.80	.53	.81
Sexual compulsivity scale	10	1.76	.61	.87
Sexual Excitation Scale	6	2.80	.55	.78
Sexual Inhibition Scale 1	4	2.27	.56	.58
Sexual Inhibition Scale 2	4	2.65	.63	.59
BASEF	15	1.96	.53	.78
BASEF (anal)	3	1.43	.64	.69
BASEF (performance)	3	2.68	.91	.56
BASEF (aging)	3	2.01	.88	.73
BASEF (pain)	3	2.07	.83	.57
BASEF (relationship)	3	1.62	.74	.65
SSCS overall mean	12	2.77	.72	.77
SSCS1 (embarrassment)	6	2.27	.96	.84
SSCS2 (self focus)	6	3.28	.78	.69
SFQ—Normophilic fantasies	27	2.87	.73	.93
SFQ—Sexualized aggression	17	1.56	.63	.93
SFQ—Sexual sumbission	14	1.59	.67	.92
SFQ—Submissive courtship	3	1.11	.37	.69
SFQ—Bodily functions	4	1.34	.60	.73
SFQ total	65	2.08	.50	.95

*BASEF* Beliefs About Sexual Functioning Scale, *SSCS* Sexual Self-Consciousness Scale, *SFQ* Sexual Fantasy Questionnaire

**Table 3:** Results of the analysis of the Sexual Fantasy Questionnaire’s nomological network (Pearson’s correlations with 95% CI)

	Normophilic fantasies	Sexualized aggression	Sexual submission	Submissive courtship	Bodily functions
*Convergent validity*					
Sexual sensation seeking	.59 [.55, .64]	.44 [.38,.49]	.20 [.14,.27]	.17 [.11,.24]	.29 [.22,.35]
Sex drive	.58 [.53, .62]	.34 [.27, .40]	.05 [−.02, .11]	.10 [.03, .16]	.24 [.18, .30]
Sexual compulsivity	.48 [.43, .53]	.45 [.40, .50]	.12 [.05, .18]	.14 [.08, .21]	.21 [.15, .28]
Sexual excitation	.55 [.50, .59]	.32 [.26, .38]	.12 [.56, .19]	.16 [.10, .23]	15 [.09,.22]
*Discriminant validity*					
Sexual Inhibition Scale 1	−.14 [−.21, −.07]	−.12 [−.19, −.06]	−.03 [−.10, .04]	.01 [−.05, .08]	−.14 [−.20, −.07]
Sexual Inhibition Scale 2	−.36 [−.42, −.30]	−.24 [−.30, −.17]	−.10 [−.16, −.03]	−.12 [−.18, −.05]	−.21 [−.28, −.15]
Sexual Embarrassment (sexual self-consciousness)	−.18 [−.24, −.11]	−.09 [−.15,−.02]	.08 [.01, .14]	.07 [.01, .14]	−.15 [.21, −.08]
Sexual Self-Focus (sexual self-consciousness)	.15 [.09, .22]	.07 [.00, .13]	.13 [.06, .20]	.04 [−.03, .10]	−.04 [−.10, .03]
Negative Beliefs about sexual function	−.04 [−.11,.03]	−.06 [−.13, .01]	−.19 [−.25, −.12]	−.00 [−.07, .06]	−.07 [−.14, −.01]

In addition, we expected small or zero correlations with additional sexuality-related measures indicative of avoidance motives for sexual behavior, which was the case for sexual inhibition, sexual embarrassment, sexual self-focus, and negative beliefs about sex. A moderate negative effect was found only for the relationship between Factor 1 (normophilic sexual fantasies) and the SIS2. In sum, the results confirm the construct validity of our version of the SFQ by showing that there are high (but no redundant) correlations with related constructs, and very low or negative correlations with distant constructs.

## Discussion

The current study contributes to the development of a reliable and valid self-report assessment of sexual fantasy use. To do so, we first tested the factorial validity of the behavioral items from the original SFQ scale by Gray et al. (2003). Second, we established a nomological network for the scale and its expected factors by testing the association of other sexuality-related measures (construct validity).

### Factorial Validity

In the present study, we aimed to test whether the proposed five-factor structure of a 59-item version of the SFQ by Bartels and Harper (2018, 2021) can be replicated in a large German sample recruited online via social media. The CFA approach was used to more rigorously examine the SFQ for use in research and to explore its potential for use in clinical practice. The five-factor structure proposed by Bartels and Harper (2018, 2021) revealed the factors Romantic (10 items), Impersonal (11 items), Sexualized Aggression (17 items), Sexualized Submission (17 items), and Bodily Functions (4 items). However, this previously proposed solution did not show a sufficient model fit in our German sample. Thus, exploratory factor analysis (EFA) was used to identify an alternative structure of the SFQ in the German sample, revealing a five-factor, 65-item scale. Three of the five factors showed substantial overlap with Bartels and Harper’s (2018, 2021) five-factor solution. Interestingly, the first factor included eight of the 10 items from the Romantic factor and all 11 items from the previous Impersonal factor found by Bartels and Harper, as well as eight additional items. Although the items “Exposing your genitals to somebody” and “Masturbating in front of another person” were included in this factor, they do not refer to an unsuspecting person. As such, we would not consider them as direct indicators of exhibitionism. Indeed, participants may have been thinking of a partner when interpreting these items. Based on this, all 27 items of this new factor seemed to predominantly assess the use of non-paraphilic (i.e., normophilic) sexual fantasies. Thus, similar to Brown et al. (2022), the factor was named accordingly. We know that, amongst others, paraphilic interest can be subdivided in relation to the “sensory channel” that is stimulated (Perrotta, 2021): the five senses (i.e., the visual channel, the acoustic/verbal channel, the olfactory channel, the gustatory channel, the tactile channel). In general, the theme of this first factor seems to be normophilic, sensory stimulation including the visual channel (e.g., “sex while watching soft pornography”) as well as the tactile channel (e.g., “touching intimate places”).

The second factor included all 17 items of the Sexualized Aggression factor found by Bartels and Harper (2018, 2021) and was named accordingly. The ‘anal sex’ item did not load on any of the factors in Bartels and Harper’s analysis, which is reasonable as it is not considered to be a core feature of sexual sadism (Marshall et al., 2002). Indeed, in the current study, the “anal sex” item loaded on the first factor, indicating this behavior to be a part of the spectrum of normophilic sexual fantasies. Also, 14 of the 17 sexualized submission items identified by Bartels and Harper (2018, 2021) loaded on a distinct factor and, thus, was named accordingly. This finding aligns with the distinction between sexual masochism and sexual sadism in the DSM-5 (APA, 2013), as well as the removal of the sadomasochism category in the ICD-11.

The last two factors each comprised items that pertained to paraphilic behavior. The fourth factor comprised three items; namely, (1) “being stalked or secretly followed by somebody”, (2) “receiving threatening phone calls”, and (3) “sex while being threatened with a weapon.” We would argue that these items are related to courtship theory (Freund & Blanchard, 1986). According to this theory, there are four phases of human courtship: (1) locating a potential partner (here, being stalked); (2) pretactile interaction (here, receiving threatening phone calls); (3) tactile interaction; and (4) copulatory interaction (here, sex while being threatened with a weapon). Given that two of the three items were part of Bartels and Harper’s (2018, 2021) sexualized submission factor, we argue that this factor constitutes a submissive variant of courtship disorder. The second group of paraphilic items included both active and passive forms of urophilia (sexual excitement caused by urination) and coprophilia (sexual excitement caused by feces/defecation), and perfectly replicated the Bodily Functions factor found by Bartels and Harper (2018, 2021). In a recent factor analysis of self-reported unusual sexual interests (Schippers et al., 2021), a similar factor emerged containing items primarily about urination and defecation, which was labeled “mysophilia, (meaning “sexual arousal to dirtiness, or soiled, decaying things,” p. 1618).

### Construct Validity: Nomological Network

Our results also supported the construct validity of the German version of the Sexual Fantasies Questionnaire, established via nomological network. Specifically, normophilic, sensory stimulation (Factor 1) and sexualized aggression (Factor 2) showed medium to large relationships with other sexuality-related measures, such as sexual sensation-seeking and sexual compulsivity. The results indicate that intensive fantasizing about these two themes is related to a need for varied, novel, and complex sexual experiences, as well as insistent and uncontrolled sexual thoughts. The same is true for sexualized submission, as well as the two paraphilic fantasy themes (submissive courtship disorder, bodily functions), albeit to a lesser degree. We would argue that these relationships can be explained in line with the Approach-Avoidance Motivation Model (e.g., Gable, 2006). Consistent with this model, we would argue that all constructs included in the nomological network (e.g., sex drive, sexual excitation) that showed (as expected) positive relationships with sexual fantasy themes, can be classified as pursuing desired outcomes, such as sexual relief (approach motivation). In line with the argument that the sexual fantasy themes indicate approach motivation in relation to sexuality, the themes showed no relationship (or small negative relationships) with motivations that can be classified as avoiding unwanted sexual outcomes or consequences (avoidance motivation). Specifically, sexual fantasy themes were not related, or were slightly negatively related, to feelings of embarrassment, uncertainty, and discomfort in sexual situations, as well as dysfunctional sexual beliefs.

### Limitations

The present results should be interpreted alongside consideration of the study’s limitations. First, the data collected were cross-sectional meaning that causal conclusions cannot be made about the association between sexual fantasizing and the other variables examined. Second, a convenience sample was collected online via social media sites, which cannot be assumed to be representative of the entire population, especially given that sexuality research is susceptible to self-selection biases (Strassberg & Lowe, 1995). Further, the sample was, on average, highly educated and largely comprised of men. As Joyal et al. (2015) notes, “young educated males are overrepresented among Internet users searching for sexual content.” In addition, we also shared the questionnaire via Reddit with men being twice as likely as women to be Reddit users (Statista, 2022). Thus, the results may be more representative of this subsection of the German population. Further research using a more representative sample is, therefore, needed to corroborate our findings. Third, sexual fantasy content cannot be objectively confirmed beyond self-report data. This can be an issue when considering that some participants may dissimulate by responding in a socially desirable manner (Bartels et al., 2019). While this may pose concerns for its use within forensic populations, sexual fantasy measures still show meaningful relationships with other risk-related variables in people who have sexually offended (Seifert et al., 2017).

It should also be noted that the range of sexual fantasies (items) was reduced after performing the factor analysis. Thus, similar to Brown et al. (2022), certain sexual interests and paraphilias are not present within the German SFQ-R, such as frotteurism and zoophilia. Those that did remain within the scale are only represented by a single item (e.g., voyeurism). It has been stated that the WSFQ contains too few items to sufficiently assess the use of paraphilic sexual fantasies (O’Donohue et al., 1997). The same can be said about this German version of the SFQ based on the current analysis. This may be due, in part, to the fact that the original scale was designed to assess sadistic sexual fantasies (Gray et al., 2003). As such, this version of the German SFQ may be more useful for those who need to assess the use of aggressive and submissive sexual fantasy content. However, the behavioral category of sexualized aggression did not reflect the within-factor variation found in other studies. For example, using a scale designed to measure aggressive sexual fantasies, Bondü and Birke (2020) found three factors reflecting slightly painful and/or consensual fantasies, coercive fantasies, and fantasies involving injury. These different aggressive themes showed differential relationships with sexual behavior (e.g., the latter two factors predicted presumably non-consensual sexual behavior). This level of specificity did not emerge in the present study as only a single aggression-related theme was found from the factor analysis. The same may also apply to the other factors (themes) found in this study.

Given that other paraphilic content is lacking in the German SFQ, which also applies to other existing scales, we argue that an entirely new measure for assessing sexual fantasizing may be needed; one that covers multiple paraphilic interests (e.g., voyeurism, exhibitionism, frotteurism, somnophilia, zoophilia, necrophilia) and multiple items for each interest. The factor structure for such a measure could then be tested to determine whether distinct factors for each paraphilic content emerge, or whether a broad factor for multiple paraphilias emerges, similar to what Dyer and Olver (2016) found. Finally, as the German SFQ focuses on behavioral content, it does not include target-specific content (e.g., children, men, women, etc.). This can be a strength, however, as it would help one to examine the behavior x target interaction (Bartels, 2020), accounting, in part, for the multifaceted nature of fantasy content (Turner-Moore & Waterman, 2023). For example, researchers or clinicians could specify the target when administering the SFQ or inquire about the fantasy target(s) after its completion. This could reveal, for example, whether content depicting specific behaviors (e.g., oral sex, touching intimate places) differs depending on the target that is envisioned, such as a partner vs. an acquaintance, or a child vs. an adult.

In conclusion, this study aimed to create a German version of Gray et al.’s (2003) SFQ that is both user-friendly and has strong psychometric properties, allowing researchers and practitioners to utilize it with confidence and ease. We were able to identify a version including 65 of the formerly 87 behavioral fantasy items. We are confident that the factors that were replicated (namely, sexualized aggression, sexualized submission, and bodily functions) can be implemented in research and practice. For example, the Sexualized Aggression and Sexualized Submission factors could allow researchers to investigate the two paraphilias separately and provide clinicians with a reliable assessment of these paraphilic interests. The first factor reflects a variation of non-paraphilic behavioral content that may be useful for determining the extent of one’s general fantasy use, especially as it showed the strongest relationship with other sexuality-related measures.
